# Spinal epidural arteriovenous fistula with nerve root enhancement mimicking myeloradiculitis: a case report

**DOI:** 10.1186/s12883-023-03097-7

**Published:** 2023-02-07

**Authors:** Sharon Chiang, Douglas B. Pet, Jason F. Talbott, Sara C. LaHue, Vanja C. Douglas, Nicole Rosendale

**Affiliations:** 1 Department of Neurology and Weill Institute for Neurosciences, University of California, San Francisco, San Francisco, CA USA; 2 Departments of Physiology and Psychiatry and the Kavli Institute for Fundamental Neuroscience, University of California, San Francisco, San Francisco, CA USA; 3 Department of Radiology and Biomedical Imaging, University of California, San Francisco, San Francisco, CA USA; 4 Buck Institute for Research On Aging, Novato, CA USA

**Keywords:** Spinal dural arteriovenous fistula, Spinal epidural arteriovenous fistula Myeloradiculopathy, Myeloradiculitis, Transverse myelitis, Nerve root enhancement, Case report

## Abstract

**Background:**

Gadolinium enhancement of spinal nerve roots on magnetic resonance imaging (MRI) has rarely been reported in spinal dural arteriovenous fistula (SDAVF). Nerve root enhancement and cerebrospinal fluid (CSF) pleocytosis can be deceptive and lead to a misdiagnosis of myeloradiculitis. We report a patient who was initially diagnosed with neurosarcoid myeloradiculitis due to spinal nerve root enhancement, mildly inflammatory cerebrospinal fluid, and pulmonary granulomas, who ultimately was found to have an extensive symptomatic SDAVF.

**Case presentation:**

A 52-year-old woman presented with a longitudinally extensive spinal cord lesion with associated gadolinium enhancement of the cord and cauda equina nerve roots, and mild lymphocytic pleocytosis. Pulmonary lymph node biopsy revealed non-caseating granulomas and neurosarcoid myeloradiculitis was suspected. She had rapid and profound clinical deterioration after a single dose of steroids. Further work-up with spinal angiography revealed a thoracic SDAVF, which was surgically ligated leading to clinical improvement.

**Conclusions:**

This case highlights an unexpected presentation of SDAVF with nerve root enhancement and concurrent pulmonary non-caseating granulomas, leading to an initial misdiagnosis with neurosarcoidosis. Nerve root enhancement has only rarely been described in cases of SDAVF; however, as this case highlights, it is an important consideration in the differential diagnosis of non-inflammatory causes of longitudinally extensive myeloradiculopathy with nerve root enhancement. This point is highly salient due to the importance of avoiding misdiagnosis of SDAVF, as interventions such as steroids or epidural injections used to treat inflammatory or infiltrative mimics may worsen symptoms in SDAVF. We review the presentation, diagnosis, and management of SDAVF as well as a proposed diagnostic approach to differentiating SDAVF from inflammatory myeloradiculitis.

## Background

Radiographic findings associated with spinal dural arteriovenous fistulas (SDAVF) are most commonly intramedullary T2 hyperintensities and venous flow voids. Gadolinium enhancement of spinal nerve roots on magnetic resonance imaging (MRI) has rarely been reported in SDAVF. Nerve root enhancement and cerebrospinal fluid (CSF) pleocytosis can be deceptive and lead to a misdiagnosis of myeloradiculitis. We report a patient who was initially diagnosed with neurosarcoid myeloradiculitis due to spinal nerve root enhancement, mildly inflammatory cerebrospinal fluid, and pulmonary granulomas, who ultimately was found to have an extensive symptomatic SDAVF. We highlight the need for improved characterization of the prevalence of nerve root enhancement as a radiographic finding in SDAVF to avoid misdiagnosis with inflammatory or infectious causes of myeloradiculopathy, and propose a diagnostic approach to differentiating SDAVF from inflammatory myeloradiculitis.

## Case presentation

**Fig. 1:**
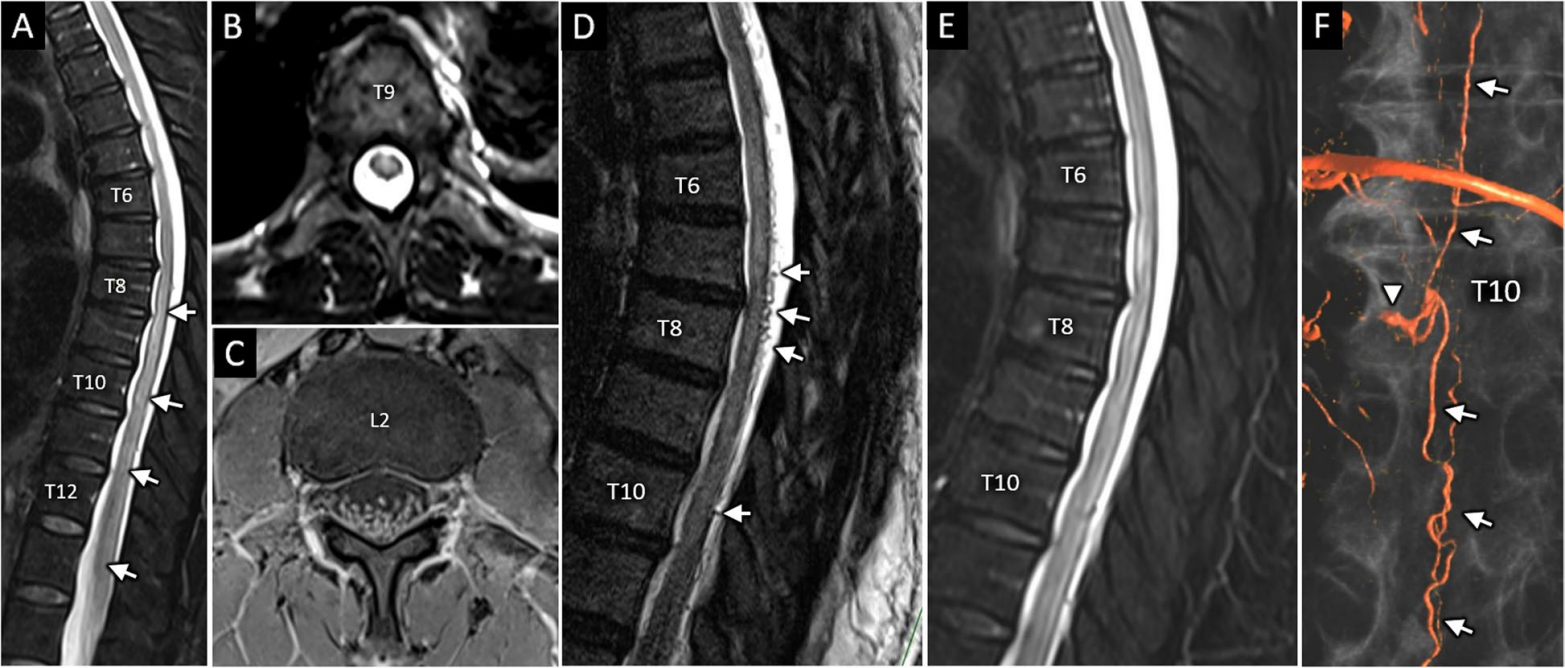
MRI characterization of SDAVF. Sagittal (**A**) and axial (**B**) T2-weighted images of the thoracic spine on day 3 reveals longitudinally extensive centromedullary T2 hyperintensity extending from T8 through the conus (A, arrowheads). Axial T1 post contrast image (**C**) at L2 shows diffuse abnormal thickening and hyperenhancement of the cauda equina nerve roots. Follow-up MRI total spine on day 32, which included high resolution sagittal 3D T2-SPACE sequence (**D**), clearly depicts numerous dilated vascular flow voids along the dorsal spinal cord surface (D, arrowheads). These abnormal vessels are not well resolved with the conventional T2 sequence (**E**) acquired as part of the same exam and registered to the same level of the thoracic spine shown in panel D. 3D volume-rendered reformatted image (**F**) from conventional digital subtraction angiogram reveals right T10 shunting AVF (arrowhead) with multiple dilated pial vessels extending craniocaudally along the spinal canal (F, arrows), confirming diagnosis of SDAVF

A 52-year-old woman with remote history of left facial nerve palsy presented with two days of progressive back pain, lower extremity paresthesias, and urinary incontinence. Her symptoms began three days prior to presentation when she awoke with severe mid-lower back pain extending to her abdomen. She subsequently developed burning paresthesias from her thighs to her toes and urinary incontinence. She presented to our institution on day 3 of symptoms, and her initial examination revealed decreased sensation to pinprick over the bilateral soles, lateral aspects of the feet, and first interspaces, as well as diminished vibratory sense in her great toes. There was trace pyramidal weakness of the left lower extremity and diffuse hyperreflexia without clonus. Ultrasound revealed bladder distension. MRI of the spine showed confluent T2 hyperintensity with patchy gadolinium enhancement in the central cord from T8 to the conus medullaris (Fig. 1A,B) and thickening and enhancement of multiple lumbosacral nerve roots (Fig. 1C). Contrasted MRI and MR angiography of the brain were unremarkable. CSF showed mild lymphocytic pleocytosis without hypoglycorrhachia, with serum and CSF studies in Table 1a. She was treated empirically with intravenous acyclovir, which was discontinued when HSV/VZV studies returned negative. CT chest with contrast showed bilateral hilar and right paratracheal lymphadenopathy. The constellation of hilar lymphadenopathy, longitudinally extensive myeloradiculopathy with MRI enhancement, and mild CSF pleocytosis raised concern for sarcoidosis. Comprehensive ophthalmologic and dermatologic examinations including skin biopsies showed no evidence of sarcoidosis or other inflammatory or infiltrative processes. Endobronchial ultrasound-guided mediastinal lymph node biopsy was performed. Her neurological exam improved throughout her hospitalization, and symptoms had resolved completely by day 13 after symptom onset. At the time of discharge, lymph node pathology results were pending and outpatient follow-up arranged.

**Table 1:** Serum and cerebrospinal fluid (CSF) testing on (**a**) initial presentation and (**b**) day 28 after presentation

	(a)	(b)
Serum	Negative studies: Anti-myelin oligodendrocyte glycoprotein antibody Anti-nuclear antibody Angiotensin converting enzyme Human immunodeficiency virus (HIV) p24 antigen HIV1/2 antibodies Cytomegalovirus PCR/IgG/IgM Epstein Barr virus (EBV) IgM Rapid plasma reagin Quantiferon Gold Lyme antibody Coccidiodes antibodies via immunodiffusion Cryptococcal antigen	Negative studies: copper, Vitamin E, folate, cryoglobulins, anti-neutrophilic cytoplasmic antibody, anti-double stranded DNA, anti-cardiolipin antibody, anti-beta-2 glycoprotein, C3/C4, and anti-Ro and anti-La
CSF	7 white blood cells × 10^9^/L (WBC) (81% lymphocytes, 16% monocytes, 3% neutrophils), 1 red blood cell × 10^9^/L (RBC) Glucose 48 mg/dL (serum glucose 113 mg/dL) Protein 73 mg/dL (normal: 15–45 mg/dL) Negative studies: CSF bacterial culture Herpes simplex virus (HSV) polymerase chain reaction (PCR) Varicella zoster virus (VZV) PCR VZV IgG Venereal disease research laboratory test	6 WBC × 10^9^/L (75% lymphocytes, 24% monocytes, 1% neutrophils) 1 RBC × 10^9^/L, glucose 87 mg/dL (serum 158 mg/dL) Protein 57 mg/dL No unique oligoclonal bands IgG Index 0.61 (normal range: 0.28–0.66) Negative studies: Cytology (repeated three times) Flow cytometry without evidence of malignancy HSV PCR VZV PCR/IgG/IgM Human T-cell lymphotropic virus antibody EBV PCR Fungal and acid-fast bacillus cultures Coccidioides antibodies via complement fixation and immunodiffusion Cryptococcal antigen Mycoplasma pneumoniae IgG/IgM West Nile virus antibody Anti-aquaporin 4 antibody Metagenomic next-generation sequencing [1]

Following her discharge, the lymph node biopsy returned showing non-caseating granulomas; however, the patient could not be reached for expedited follow-up to address these findings. On day 27 after symptom onset, she presented to follow-up clinic reporting ten days of recurrent lower extremity weakness and numbness, urinary incontinence, and new constipation. Examination in clinic showed bilateral iliopsoas weakness (4/5 MRC scale) with intact distal strength, decreased sensation over the bilateral thighs and lower abdomen, and independent ambulation with unsteady gait. She was re-admitted for suspected neurosarcoid myeloradiculitis. Examination several hours later showed progression of her lower extremity weakness to 3/5 MRC strength in the bilateral iliopsoas and new pyramidal weakness (4/5) in the distal lower extremities. A T4 sensory level was appreciated and deep tendon reflexes were now absent at the left Achilles and patella. Anal wink was absent and sphincter tone was diminished. Within twelve hours of admission, she had lost the ability to stand or ambulate without two-person assistance. Repeat MRI of the spine showed radiographic progression from 24 days prior with increased intramedullary T2 hyperintensity extending from T4 to the conus medullaris. Again noted was patchy enhancement of the ventral cord and diffuse thickening and enhancement of multiple thoracolumbar nerve roots.

She was started on intravenous methylprednisolone. Twelve hours after her first dose, her examination had worsened to near-complete paraplegia with only trace muscle activation in the proximal lower extremities, as well as diffuse hyperreflexia with sustained ankle clonus bilaterally. Repeat CSF and serum analysis on day 28 are in Table 1b. Clinical deterioration following steroid administration raised concern for occult infection or SDAVF. Prior spinal imaging was reviewed to interrogate the working diagnosis of sarcoidosis and look for subtle evidence of SDAVF; no nodularity, subpial enhancement, or venous flow voids were appreciated. Ultimately, concern remained for neurosarcoid myeloradiculitis given prominent nerve root involvement and enhancement and tissue evidence of pulmonary sarcoidosis.

She received five days of intravenous methylprednisolone followed by oral prednisone 60 mg daily. Her examination improved gradually over several days with increased lower extremity activation noted by day 32 after symptom onset and subsequently to 3–4/5 MRC strength by day 35. MRI of the lumbar and thoracic spine was repeated on day 32 with thin sagittal sections using high-resolution 3D T2-weighted *S*ampling *P*erfection with *A*pplication optimized *C*ontrasts using different flip-angle *E*volutions (SPACE) sequences, which showed abnormal flow voids along the dorsal spinal cord surface from T5-T12 (Fig. 1D). This finding, occult on standard T2 sequences (Fig. 1E) and appreciated only on SPACE sequences, increased concern for SDAVF and prompted spinal angiogram on day 35, which showed a right T10 epidural arteriovenous fistula supplied by ipsilateral T9 and contralateral T10-11 intercostal arteries (Fig. 1F). Steroids were discontinued. The fistula was not amenable to endovascular treatment and she underwent surgical ligation on day 41. Post-operative angiogram showed no residual fistula. She was discharged on day 50 to a rehabilitation facility for ongoing physical therapy. Upon hospital discharge, she could stand with assistance, and lower extremity strength had improved to 3/5 in iliopsoas, 4–5/5 in quadriceps/hamstrings, and 5-/5 in tibialis anterior. She underwent repeat spinal angiogram four months later that did not reveal any residual fistula. She was seen again in clinic 10 months following surgery. At this appointment, she had regained full strength in her legs, but continued to experience numbness from her bilateral knees extending to her feet, which limited her ability to walk, necessitating use of a walker. She underwent repeat MRI 12 months after her initial surgery that demonstrated decreased conspicuity and size of the long segment T2 signal abnormality throughout the thoracic spine.

## Discussion and conclusions

SDAVF is an important consideration in the differential diagnosis of myeloradiculopathy, which includes infectious, autoimmune, neoplastic, paraneoplastic, metabolic, toxic, and other vascular etiologies. SDAVF disproportionately affects men with a male-to-female ratio of 5:1 and mean age of 55–60 years [2]. Although its exact pathophysiology is unknown, SDAVF is believed to be an acquired disease in which an aberrant connection forms between a radicular artery and vein within the dura or nerve root sleeve. Arterialization of radicular veins increases hydrostatic pressure within local venous plexuses resulting in cord edema. Direct compression from dilated veins may also result in cord/root compression [3, 4]. The majority of SDAVF are thoracolumbar and extend through multiple spinal segments, with venous dilation spanning a mean of 10 ± 7.7 levels [5]. Spinal angiography is diagnostic and therapeutic in cases amenable to embolization, while others require neurosurgical coagulation or excision.

SDAVF is challenging to diagnose, with an average time of symptom onset to diagnosis ranging 11–27 months [6]. Initial symptoms are often non-specific and involve motor, sensory, gait, and bowel/bladder disturbances that can mimic not only other forms of myelopathy, but also polyradiculopathy, peripheral neuropathy, or cauda equina syndrome [2, 7]. Characteristic intramedullary T2 hyperintensities and venous flow voids on spinal MRI are seen in 89–92% and 81% of cases, respectively [8]. However, as this case illustrates, flow voids may not be present on standard MRI sequences and their visualization may require thin-slice “myelographic” MRI techniques such as 3D T2-spin echo (i.e.. SPACE or CUBE) or balanced steady-state free precession (i.e. CISS or FIESTA). Venous congestion can also be seen as deoxyhemoglobin, which appears as linear T2 hypointensities. Nerve root involvement with or without associated enhancement in SDAVF is underrecognized in existing literature. Although approximately 80% of SDAVF involves the conus medullaris [9], the prevalence of nerve root involvement/enhancement has only rarely been described [7, 10]. SDAVF-associated root enhancement, especially if accompanied by CSF pleocytosis, can be misleading by suggesting an inflammatory or infiltrative rather than vascular process. CSF is often bland; however, mild lymphocytic pleocytosis (often up to 12/μL, although up to 93/μL has been reported) [11, 12] and/or protein elevation can be seen. CSF findings are hypothesized to result from increased intravenular pressure and transudative flow of protein and cells into CSF. CSF glucose is usually normal, although mild hypoglycorrhachia has been reported [13]. We encountered an especially rare case of SDAVF presenting with nerve root enhancement, mildly inflammatory CSF, and non-caseating granulomas. This case highlights the need for improved characterization of the prevalence of nerve root enhancement as a radiographic findings in SDAVF.

**Fig. 2:**
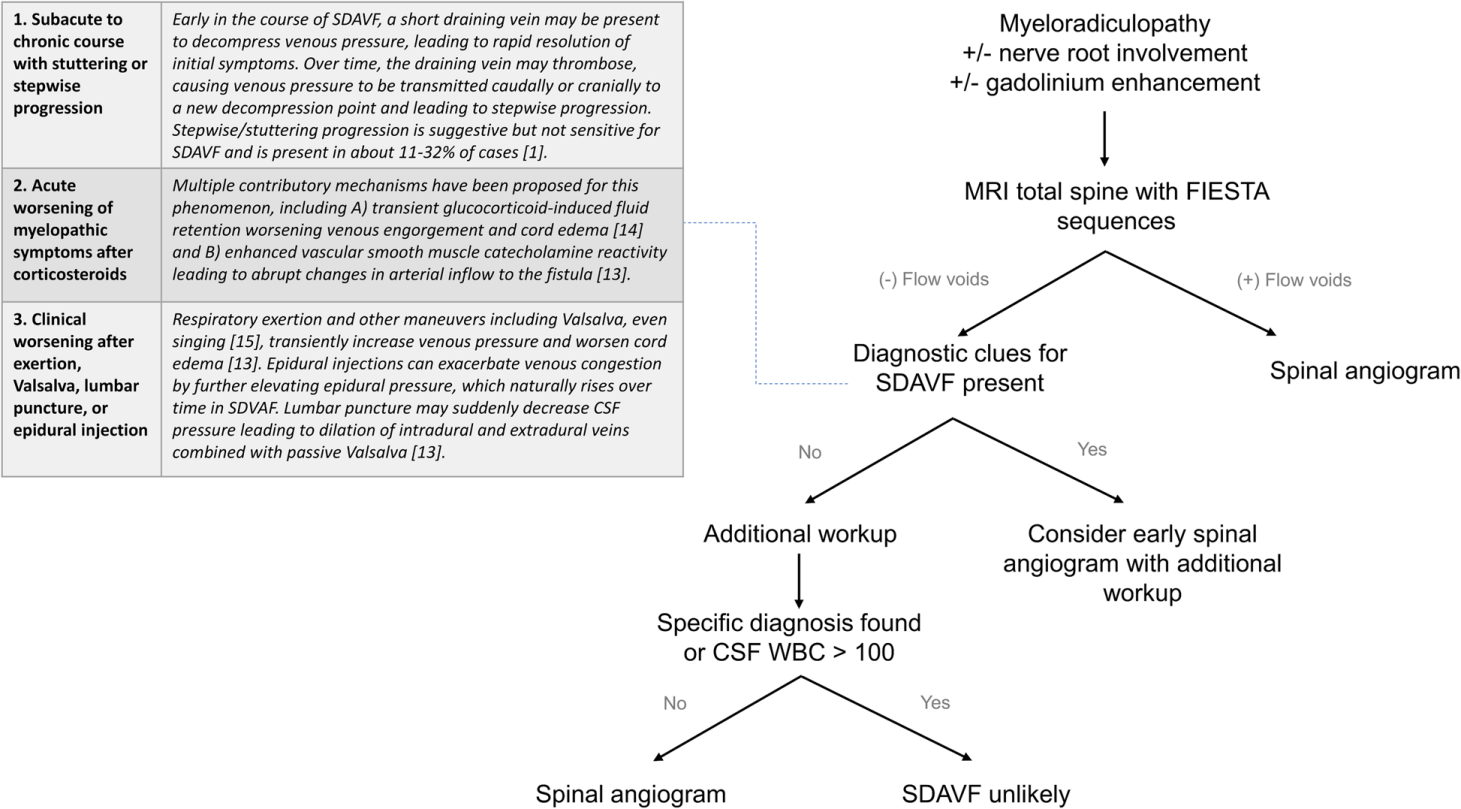
Diagnostic imaging of longitudinally extensive myeloradiculopathy when considering SDAVF

This case also demonstrates that MRI with high-resolution 3D T2 spin echo sequences such as SPACE/CUBE or balanced steady-state free precession sequences such as CISS/FIESTA should be considered in all cases with longitudinally extensive myeloradiculopathy, and it is important to recognize that nerve root enhancement can be a feature of non-inflammatory etiologies. Additional clinical features that heighten suspicion for SDAVF when both inflammatory and non-inflammatory etiologies are on the differential include a subacute-to-chronic course with stepwise progression, periods of spontaneous improvement, and worsening after corticosteroids [14]. The diagnosis may also be suggested if symptoms worsen after exertion, Valsalva [15], lumbar puncture, or epidural injections[14]. Presence of these clinical features increases pretest probability of SDAVF and is useful for informing decisions about whether certain treatments (e.g., corticosteroids) should be deferred until SDAVF has been excluded. Figure 2discusses a proposed diagnostic approach to differentiating SDAVF from inflammatory myeloradiculitis. Avoiding misdiagnosis of SDAVF through such a systematic approach is especially important given that these common treatments for SDAVF-mimics may worsen symptoms and disability in SDAVF [14].

## Data Availability

Data sharing is not applicable to this article as no datasets were generated or analyzed during the current study.
